# Detection of rabies virus *via* exciton energy transfer between CdTe quantum dots and Au nanoparticles

**DOI:** 10.3389/fvets.2022.1079916

**Published:** 2022-12-22

**Authors:** Yan-Juan Liao, Duo-Duo Li, Zong-Ling Cai, Ying Cao, Dong-Ling Yu, Hong-Yun Zhang, Abraha Bahlbi Kiflu, Zai Yin Huang, Xiao-Ning Li, Ting Rong Luo

**Affiliations:** ^1^State Key Laboratory for Conservation and Utilization of Subtropical Agro-Bioresources, Guangxi University, Nanning, Guangxi, China; ^2^Guangxi Marine Microbial Resources Industrialization Engineering Technology Research Center, Nanning, Guangxi, China; ^3^Key Laboratory of Protection and Utilization of Marine Resources, Guangxi University for Nationalities, Nanning, Guangxi, China

**Keywords:** detection, rabies virus, signal amplification strategy, quantum dots (DQs), photoelectrochemical biosensing approach

## Abstract

Rabies is a fatal encephalitis caused by the rabies virus. The diagnosis of the disease depends in large part on the exposure history of the victim and clinical manifestations of the disease. Rapid rabies diagnosis is an important step in its prevention and control. Therefore, for accurate and timely diagnosis and prevention of rabies, we developed nanomaterials for a novel photoelectrochemical biosensing approach (PBA) for the rapid and reliable diagnosis of rabies virus. This approach uses high-efficiency exciton energy transfer between cadmium telluride quantum dots and Au nanoparticles and is low cost, and easy to miniaturize. By constructing PBA, rabies virus can be detected quickly and with a high degree of sensitivity and specificity; the minimum detection concentration limit for rabies virus is approximately 2.16 ffu/mL of rabies virus particles, or 2.53 × 10^1^ fg/mL of rabies virus RNA. PBA could also detect rabies virus in the brain and lung tissue from rabid dogs and mice with better sensitivity than RT-PCR.

## 1. Impact statement

Herein, we used a new photoelectrochemical biosensing approach based on a signal amplification strategy to detect the rabies virus. This method uses high-efficiency exciton energy transfer between cadmium telluride quantum dots (CdTe QDs) and Au nanoparticles, and the photocurrent intensity increases dramatically if rabies virus is present. To the best of our knowledge, this is the first report on rabies virus detection methods using this strategy, and the detection limit for rabies virus concentration was estimated to be 2.16 ffu/mL, or 2.53 × 10^1^ fg/mL. PBA could also detect rabies in brain and lung tissue from rabid dogs and mice with better sensitivity than RT-PCR.

## 2. Introduction

Rabies is caused by the rabies virus (RABV), which infects the central nervous system of almost all mammals with a mortality rate of nearly 100% (1). Although several effective and fast diagnostic methods have been used to detect RABV (2–4), current methods are limited by the low abundance of RABV in brain tissue and/or saliva samples.

Current detection methods include conventional and nucleic acid diagnostic methods, the direct fluorescent antibody test (DFAT), and the mouse inoculation test (MIT). DFAT has high sensitivity and specificity but requires well-trained personnel and an expensive fluorescence microscope (5). The most important disadvantage of MIT is its delayed results (up to 28 days) and the need for animal facilities and adequate containment (6). Direct rapid immunohistochemical test (DRIT) is a diagnostic method for detecting RABV by biotin-labeled anti- RABV monoclonal antibody combined with RABV antigen (7).

Viral RNA can be efficiently detected by PCR-based methods such as reverse transcription polymerase chain reaction (RT-PCR) (8), real-time RT-PCR (qRT-PCR) (9), and hemi-nested RT-PCR (10). The N gene has been identified as a diagnostic marker (11) because it is the most conserved region in the RABV genome. Although amplification methods are widely used, they often lead to false-negative results because of the single sequence mismatch between the primer or probe sequence designed according to the N gene; the target viral sequence may also alter the sensitivity of the test (12, 13), or the different kits used for qRT-PCR detection are prone to yield variant-dependent false-negative test results (14).

Photoelectrochemical techniques are an emerging class of powerful analytical methods for biological research. Compared to electrochemical and optical methods, photoelectrochemical detection offers simple devices, low cost, and easy miniaturization (15). Photoelectrochemical sensors combined with other RNA detection methods, such as combining with CRISPR method, to detect RNA (16). Moreover, this strategy uses different forms of energy for excitation and detection; thus, there is less background signal and improved sensitivity (17). Examples include the identification of early-stage cancer cells, characterization of biological processes, identification of small-molecule functions (18), and rapid detection of SARS-CoV-2 (19).

Two necessary devices for constructing current-type photoelectrochemical sensors include biometric elements (photoelectric active materials) and biometric probes. When the current-type photoelectrochemical sensor is excited by light, the photocurrent can be produced by photogenerated electrons transferred from the photoelectric active material to the electrode of the sensor. The specificity of the corresponding target analytes can be evaluated *via* a change in photocurrent. The photoelectric active material is a core factor. The biometric probes are usually protein or nucleic acids. Photocurrent signal generation is the main goal for improving photoelectric active materials. Photocurrent generated by energy transfer is an important type of sensor (20, 21). Energy transfer is a particularly powerful analytical method for the sensitive detection of various biomolecules under suitable conditions between energy donors and acceptors. The energy excited by the energy donor is transferred to the adjacent energy receptor. Proximal acceptors capture exciton energy by photoactive donors through non-radiative dipole-dipole interactions, which is a non-radiative process (22). Energy transfer in this type of sensor needs to satisfy two conditions simultaneously. First, the distance between the energy donor and the energy receptor is 10 ± 2 nm. Second, there should be a spectral overlap between the emission spectrum of the energy donor and the absorption spectrum of the energy receptor (23).

Here, an enhanced photoelectrochemical aptamer-sensing platform was constructed for rabies virus detection using classical semiconductor cadmium telluride quantum dots (CdTe QDs) with good light stability, a wide excitation range, and an adjustable emission spectrum as energy donors; AuNPs served as the energy transfer receptors (22). CdTe QDs have tunable emission spectra *via* the Cd:Te ratio and reaction conditions. The absorption spectra of the AuNPs were regulated by controlling the HAuCl_4_ concentration and the ratio of sodium citrate to NaBH_4_. This photoelectrochemical aptamer sensing platform led to specific, fast, and sensitive rabies virus detection (24).

## 3. Materials and methods

### 3.1. Experimental materials and reagents

Cadmium chloride (CdCl_2_·2.5H_2_O), sodium tellurite (Na_2_TeO_3_), chloroauric acid (HAuCl_4_·4H_2_O), Tris (2-Chloroethyl) phosphate (TCEP) were obtained from Aladdin Reagent Inc. (China). Trisodiumcitrate dihydrate TiO_2_), N-hydroxysuccinimide (NHS), 1-ethyl-3-(3-dimethylaminopropyl) carbodiimide hydrochloride (EDC), monoethanolamine (MEA), 3-mercaptopropionic acid (MPA), and 6-mercaptohexanol (MCH) were obtained from Sigma-Aldrich (USA). Indium tin oxides (ITO) electrodes (type JH52, ITO coating 30 ± 5 nm, sheet resistance ≤ 10 Ω/square) were obtained from Beijing Zhongjingkeyi Technology Co., Ltd. (China). All aqueous solutions were prepared from deionized water (DI water, 18 MΩ/cm) using a Milli-Q water purification system. RNA solution, washing buffer solution, and blocking buffer solution (containing 1 mM MEA) were prepared using a phosphate buffer solution (PBS, pH7.4, 20 mM). Total cellular RNA was extracted using TRIzol (Invitrogen, Shanghai, China) from the cell culture, then the RNA simple Total RNA Kit (purchased from Tiangen Biotech Co, Beijing, China) was used to purify the RNA further. Synthetic reagents of first-strand complementary DNA (cDNA) which include Moloney murine leukemia virus (M-MLV) reverse transcriptase, recombinant RNase Inhibitor and dNTP Mixture were purchased from Takara Company (Takara, China).

Synthetic oligonucleotides with the following sequences were obtained from Shenggong Bioengineering Co., Ltd. (Shanghai, China): The probe sequence was designed according to the N gene of RABV. The uni- 98 versal probe of RABV (pRNA, NH_2_-RNA-SH): 5′-SH-(CH_2_)_3_-TTT ACC ATA CGG CCG GGC AAT CTG AAG TTC GGT ATG GT-(CH_2_)_6_-NH_2_-3'. The r-RC-HL RABV probe (p-r-RNA) was 5′-SH-(CH_2_)_3_-TTT ACC ATA CGG CCG GGC AAG CAG YGA CAA CAG TAC C-(CH_2_)_6_-NH_2_-3'; the GX074 wild RABV probe (p-w-RNA) was 5′-SH-(CH_2_)_3_-TTT ACC ATA CGG CCG GGC AAT AGG AAT GAG GAA CAG C-(CH_2_)_6_-NH_2_-3'. The CVS-N2C RABV probe (p-c-RNA) was 5′-SH-(CH_2_)_3_-TTT ACC ATA CGG CCG GGC ATG TTT GTC TTG TAG TTG C-(CH_2_)_6_-NH_2_-3'; primers for reverse transcription and PCR were N1: (5′-ACAGACAGCGTCAATTGCAAAGC-3′) and N2: (5′-CAGTCTTCATAAGCAGTGACAAC-3′).

### 3.2. Equipment

Photoelectrochemical measurements were performed using a homemade photoelectrochemical system. A 500-W Xe lamp with a spectral range of 200–2500 nm was used as the irradiation source with a light intensity of 400 μW·cm^−2^ as measured by a radiometer (Photoelectric Instrument Factory of Beijing Normal University). The photocurrent was measured on a CHI 660D electrochemical workstation (Shanghai Chenhua Apparatus Corporation, China). The UV-visible (UV-vis) absorption spectra were recorded on a UV-3600 UV-visible spectrophotometer (Shimadzu, Japan). Photoluminescence (PL) spectra were recorded on a RF-5301PC spectrofluorometer (Shimadzu, Japan). Field-emission scanning electron microscopy and transmission electron microscopy were used to observe the synthesized nanomaterials.

### 3.3. Synthesis of AuNPs

Water-soluble AuNPs were synthesized using a previously reported method with minor modifications (25). First, 50 μL of 100 mM HAuCl_4_ and 5.0 mL of 10 mM sodium citrate were mixed gradually in 75 ml DI water in a round-bottomed flask under continuous stirring. Next, 50 μL of 0.1 M ice-cold NaBH_4_ was quickly injected into the solution, which turned orange-red, indicating particle formation. The mixed solution was then stirred for another 6 h at room temperature with a color change from orange-red to wine-red. Finally, the resulting AuNPs solution was obtained and stored at 4°C before use.

### 3.4. Synthesis of AuNPs-pRNA conjugates

First, 280 μL of 10 μM RNA probe (pRNA) was activated by adding 10 μL of 10 mM Tris(2-Chloroethyl)phosphate (TCEP) for 1 h to break the disulfide bond between the sulfhydryl-functionalized pRNA. Next, 1000 μL of purified AuNPs solution was added to the pRNA solution and incubated for 12 h with shaking in the dark at 20°C. To prevent nonspecific adsorption of the AuNPs, 50 μL of 0.1 mM MCH was injected and incubated for 2 h under shaking. The resulting AuNPs-pRNA conjugates were acquired from the supernatant after the resulting mixture was centrifuged at 5000 rpm 4 min each time, for 3 times.

### 3.5. Fabrication of TiO_2_:C-NPs/ITO electrode

Before use, bare ITO slices were ultrasonically cleaned by the ultrasonic cleaner (Reliance Digital, England) with an ultrasound frequency of 42 kHz with acetone, 1.0 M NaOH ethanol/water mixture (1:1, v/v), and DI water for 15 min. The samples were then dried at 90°C. TiO_2_:C-NPs powder (4.0 mg) was ultrasonically dispersed in 2.0 mL of deionized water, and 20 μL of uniformly dispersed TiO_2_:C-NPs suspension (2.0 mg/mL) was drip-coated on an ITO electrode with a modified area of 0.25 cm^2^. After drying at room temperature, the sample was calcined in air at 450°C for 30 min, and then slowly cooled to room temperature to obtain the resulting TiO_2_:C-NPs/ITO electrode.

### 3.6. Synthesis of CdTe QDs

Water-soluble COOH-capped CdTe QDs were synthesized as described (26). Typically, 0.6 mmol CdCl_2_·2.5H_2_O and 1.02 mmol MPA were mixed in a three-necked flask with constant stirring. Thereafter, the pH of the solution was adjusted to 11.8 by dropwise addition of 1.0 M NaOH, and the solution was bubbled with pure nitrogen gas for 30 min. Afterwards, 0.06 mmol Na_2_TeO_3_ and 120 mg NaBH_4_ were successively added to the flask at a typical molar ratio of Te^2−^: Cd^2+^:MPA of 0.1:1:1.7. Finally, the desired CdTe QDs were obtained by heating at 100°C and refluxing for 3 h under nitrogen.

### 3.7. Preparation of TiO_2_:C-NPs/CdTe electrode

The modification of CdTe QDs adopts a layer-by-layer self-assembly method. First, the TiO_2_:C-NPs electrode was immersed in the CdTe QDs solution (20 μL) for 10 min and then cleaned with ultrapure water. The two-step immersion was used as a modified “layer” of CdTe QDs. The required TiO_2_:C-NPs/CdTe electrode was obtained after modifying the four layers.

### 3.8. Preparation of Au/SH-PRNA conjugates

AuNPs were resuspended in 1 mL of 10 μM SH-RNA activated with TCEP (10 mM) and shaken for 12 h. The unbound SH-PRNA (SH-PRNA unlinked to AuNPs) was removed *via* 15 min of centrifugation at 4°C, 5000 rpm for twice, resulting in Au/SH-RNA.

### 3.9. Aptasensor fabrication

The NH_2_-RNA-SH-AuNPs were immobilized onto the TiO_2_:C-NPs/CdTe electrode *via* the classic EDC coupling reaction between the carbonyl groups on the surface of the MPA-capped CdTe QDs and the amino groups of the NH_2_-RNA-SH-AuNPs. First, the CdTe QDs-modified electrode was activated by adding 25 μL DI water containing 20 mM EDC and 10 mM NHS for 1 h at room temperature followed by rinsing with Tris-HCl buffer (10 mM, pH 7.4) to remove the excess EDC and NHS. Next, 20 μL of 1 μM NH_2_-RNA-SH-AuNPs was dropped onto the electrode surface and incubated at 4°C overnight. The electrode was then rinsed with Tris-HCl buffer to remove unbound NH_2_-RNA-SH-AuNPs. The electrode was then blocked with 20 μL of 1 mM MEA at room temperature for 1 h and thoroughly rinsed with Tris-HCl buffer and the aptasensor has been Fabricated.

### 3.10. Sample preparation

Porcine samples infected with classical swine fever virus (CSFV), porcine reproductive and respiratory syndrome virus (PRRSV-5, PRRSV-96), porcine circovirus (PCV), and the vesicular stomatitis virus glycoprotein gene (VSV-g) were collected and preserved in our laboratory. Clinical dog samples (containing brain samples DB1, DB2, DB3, and DB4 as well as lung sample DL1) were obtained from rabid dogs between 2019 and 2021 from Guangxi province, provided by the Guangxi Center for Animal Diseases Control and Prevention with permission from the Veterinary Administration of the Guangxi Provincial Government. Kunming mice were purchased from the Animal Centre of Guangxi Medical University. The positive samples of mice were mainly inoculated with the standard strain of rabies virus (CVS-24), the dosage was 100 folds of the LD_50_, and a volume of 30 μL was injected into the brain of each mouse, and the brain samples and lung samples on the 5th day after the challenge were collected (denoted as: MB1, MB2; ML1, ML2) for detection. To comply with the Animal Research: Reporting *in Vivo* Experiments (ARRIVE) guidelines, all husbandry and experimental procedures were conducted in compliance with the Animal Welfare Act and the Guide for the Care and Use of Laboratory Animals. The mice were euthanized in a container after administration of inhaled halothane with the container closed once the anesthetized mouse displayed a lack of righting reflex. Sample detection was completed in the biological safety laboratory of Guangxi Center for Animal Diseases Control and Prevention.

### 3.11. Mouse inoculation test

The dog brain tissue (DB1, DB2, DB3, DB4) and mouse brain tissue (MB1 and MB2), were grinded into 10% emulsion (w/w) with PBS. Samples were centrifuged at 5000 rpm for 10 min, then the supernatant was inoculated intracranially in 20 ± 2 day-aged mice, with a volume of 30 μL virus solution to each mouse, and 4–6 mice were inoculated per specimen. The symptoms of mice were recorded three times per day.

### 3.12. Reverse transcription polymerase chain reaction detection

The brain tissue sample was ground with liquid nitrogen and then lysed by adding TRIzol lysis buffer to a ratio of 1:10 brain sample emulsion. The total RNA was extracted according to the instructions of the total RNA extraction kit. The reverse transcription reaction system was composed of reverse transcription 10 μM primer (N1) 1 μL, 200 U/μL reverse transcriptase (M-MLV) 0.5 μL, 40 U/μL RNase inhibitor (RNasin) 0.5 μL, 5 × Buffer 5 μL, 10 Mm dNTP 2 μL, and RNA 16 μL (1.0 μg of total RNA). The temperature program was 42°C for 1 min, 16°C for reverse, and 75°C for 5 min. The PCR reaction system was composed of 10 μM upstream primer (N1) 0.5 μL, 10 μM downstream primer (N2) 0.5 μL, 5 U/μL 2 × Taq Mix 12.5 μL, ddH_2_O 8.5 μL, and cDNA 3 μL. The process was performed at 95°C for 5 min, 95°C for 40 s, 58°C for 40 s, and 72°C for 1 min (for 30 cycles), followed by 2°C for 5 min and 16°C for preservation.

### 3.13. Photoelectrochemical biosensing approach

The mortar and pestle were autoclaved in advance and kept at −20°C before use. Then the brain tissue sample was weighted (1~2 g) and liquid nitrogen was added to grind the sample in to powder. Later 0.1 g of the tissue sample was lysed by adding 800 μL of TRIzol lysis buffer for RNA extraction. The cell samples were lysed with liquid nitrogen, and then thawed at 37°C for 5 min, and the process of freezing and thawing was repeated for three cycles. To ensure all viral RNA releasing from cells. Finally, 20 μl of cell sample or brain sample was taken and incubated with a photoelectrochemical aptasensor at 37°C for 1 h. The electrode was rinsed with Tris-HCl buffer and prepared for PBA detection.

PBA detection was performed at room temperature in Tris-HCl buffer (0.1 M, pH 7.4) containing 0.1 M ascorbic acid (AA), which served as a sacrificial electron donor during photocurrent measurements. Light excitation (460 nm) was switched on and off every 10 s. The applied potential was 0.0 V. The AA electrolyte was decreased using pure nitrogen for 15 min before the photocurrent was measured. Each sample was measured five times.

## 4. Results

**Scheme 1:**
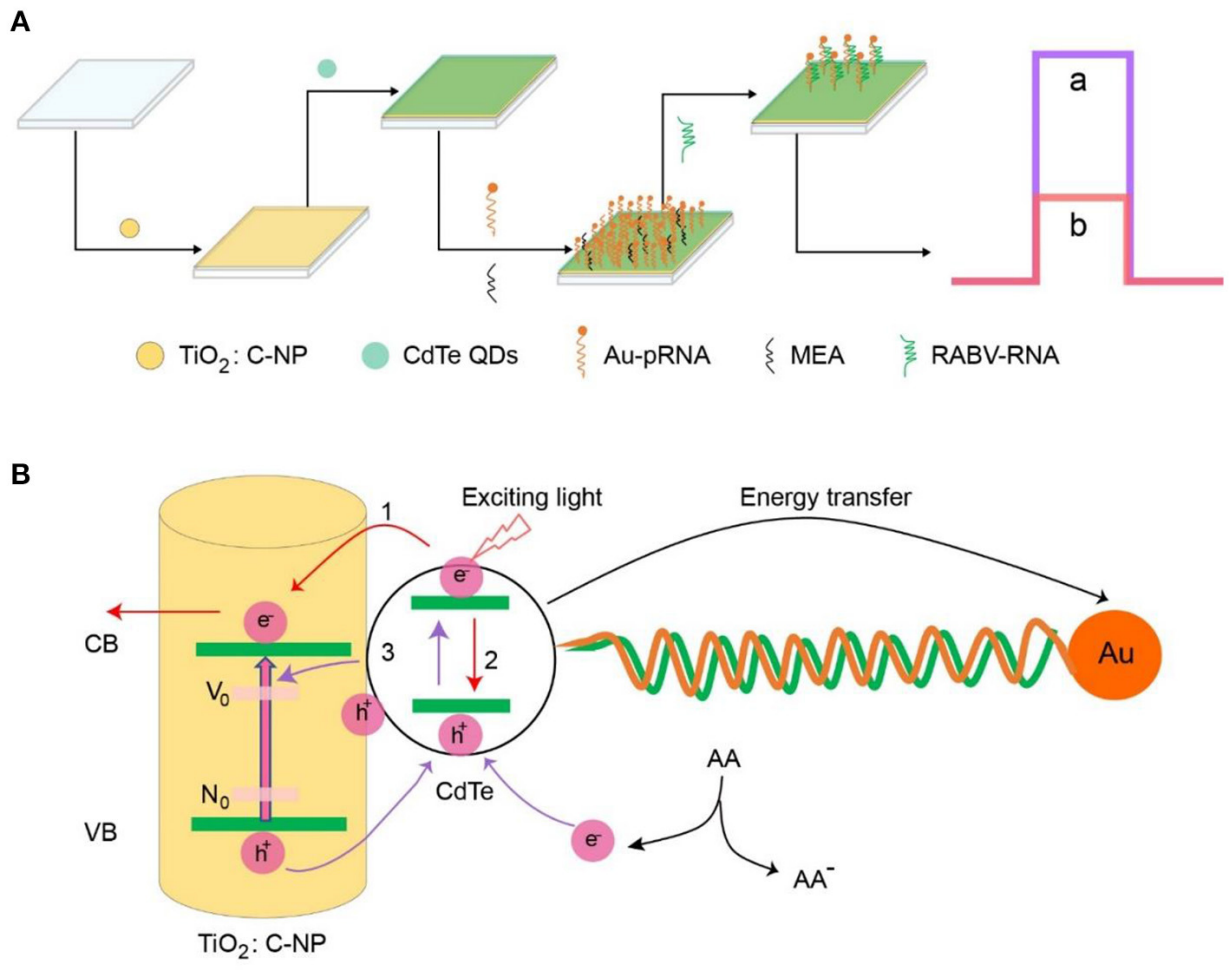
**(A)** Fabrication of the photoelectrochemical aptasensor. **(B)** Photoelectrochemical mechanism of the aptasensor.

The RABV was the target analyte and was detected *via* an enhanced photoelectrochemical aptamer sensor using CdTe QDs as energy donors and AuNPs as energy acceptors (Scheme 1A). First, CdTe QDs were assembled on the surface of a TiO_2_:C-NPs electrode *via* electrostatic adsorption. The NH_2_-RNA-SH/AuNPs were immobilized on a TiO_2_:C-NPs/CdTe electrode *via* a classical EDC coupling reaction between the carbonyl groups of CdTe QDs and the amino group of NH_2_-RNA-SH/AuNPs. This led to an aptamer sensor of TiO_2_:C-NP/CdTe/AuNPs/pRNA. MEA was then used to block the unbound sites on the electrode surface to avoid non-specific binding and false positives.

The photoelectrochemical mechanism of the aptamer sensor is shown in Scheme 1B. TiO_2_:C-NPs and CdTe QDs were excited under light conditions to generate electron-hole pairs (excitons), and photogenerated electrons transition to the conduction band (CB); holes are in the valence band (VB) (27, 28). The photogenerated electrons of CdTe QDs have two flow directions: the electrons flow to TiO_2_:C-NPs to enhance the photocurrent intensity of the sensor (Scheme 1B, process 1) while electrons flow back to the holes, thus promoting the recombination of electron-hole pairs and weakening the intensity of the photocurrent of the sensor (Scheme 1B, process 2).

These two processes have a competitive relationship. The C doping can provide a low-energy impurity level for the electron transition of TiO_2_ NPs, thus promoting photogenerated electron-hole separation and increasing electron flow in TiO_2_ NPs, leading to the generation of CdTe QDs. The photoluminescence (PL) of CdTe QDs can be absorbed by AuNPs *via* surface plasmon resonance (SPR) leading to energy transfer. The local electric field around the AuNPs increases the recombination probability of electron-hole pairs in the CdTe QDs and reduces the sensor photocurrent. The sensor photocurrent can thus be regulated by the direction of electron transfer *via* the exciton energy transfer effect. Fast and efficient exciton energy transfer between CdTe QDs and AuNPs is achieved when the sensor can no longer combine with the rabies virus. That is, the photocurrent decreases rapidly by the electron hole pair (process 2) and the inhibition (electron transfer; process 1). The aptamer was specifically bound to RABV when RABV was added to the electrode system; the exciton energy transfer effect was destroyed after Au/SH-RNA detachment from the layer of the electrode. The photocurrent intensity increased dramatically from b to a (Scheme 1A). Therefore, the rabies virus analyte can be detected sensitively by changing the photocurrent value through specific binding with the probe.

**Figure 1:**
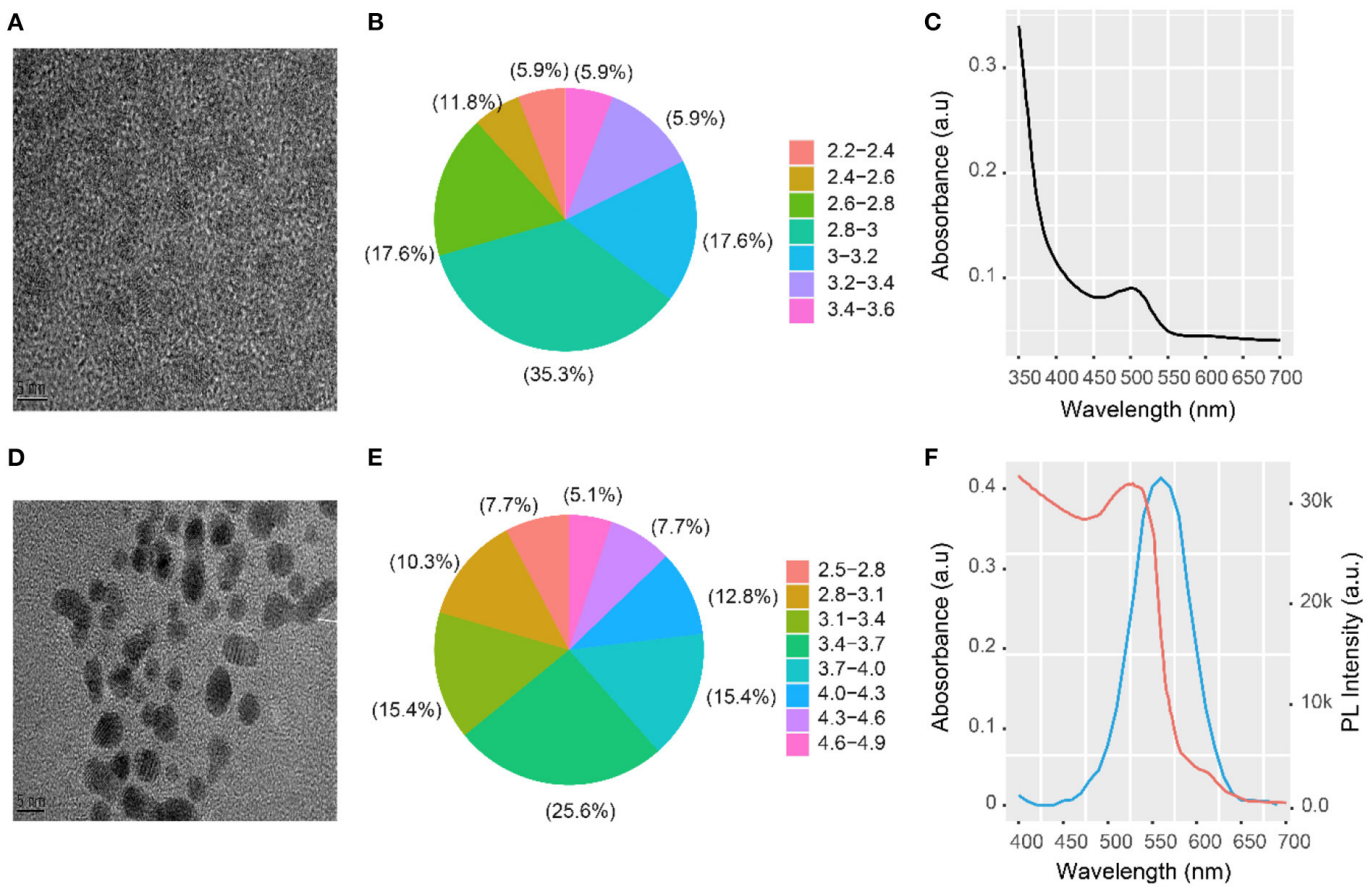
HRTEM image and UV-vis absorption spectrum of CdTeQDs and AuNPs. **(A)** HRTEM image, **(B)** size distribution, and **(C)** UV-vis absorption spectrum of CdTe QDs; **(D)** HRTEM image and **(E)** size distribution of AuNPs; **(F)** PL emission spectrum of CdTe QDs (blue curve) and absorption spectrum of the AuNPs (red curve) along with UV-vis absorption spectrum of CdTe QDs.

CdTe QDs and AuNPs were successfully synthesized, as shown in Figures 1A,D. The size distribution of the CdTe QDs and AuNPs was also measured, and their average particle sizes were 2.93 ± 0.53 nm (Figure 1B) and 3 ± 0.62 nm (Figure 1E), respectively. This is consistent with the UV-Vis absorption data and relationship of Peng et al. (29). The UV-Vis absorption peak of the CdTe QDs was 510 nm (Figure 1C). The PL emission of the CdTe QDs overlaps with the absorption spectrum of the AuNPs—this is an important condition for exciton energy transfer. Figure 1F shows the emission spectrum curve of CdTe QDs with an emission peak at 560 nm (Figure 1F, blue curve). The red curve is the absorption curve of AuNPs with a peak at 538 nm (Figure 1F, red curve). The blue and red curves have a wide overlapping area that meets the necessary conditions for efficient energy transfer.

The second condition for efficient energy transfer is that the distance between the energy transfer donor CdTe QDs and the acceptor AuNPs should be < 10 nm. Here, the distance between the energy transfer donor and the receptor was the length of the NH_2_-RNA–SH–AuNPs. The chain had 38 bases, and its length was calculated to be ~5 nm. All the conditions required for energy transfer were met. Therefore, the constructed energy transfer sensor could be used for subsequent detection and analysis.

**Figure 2:**
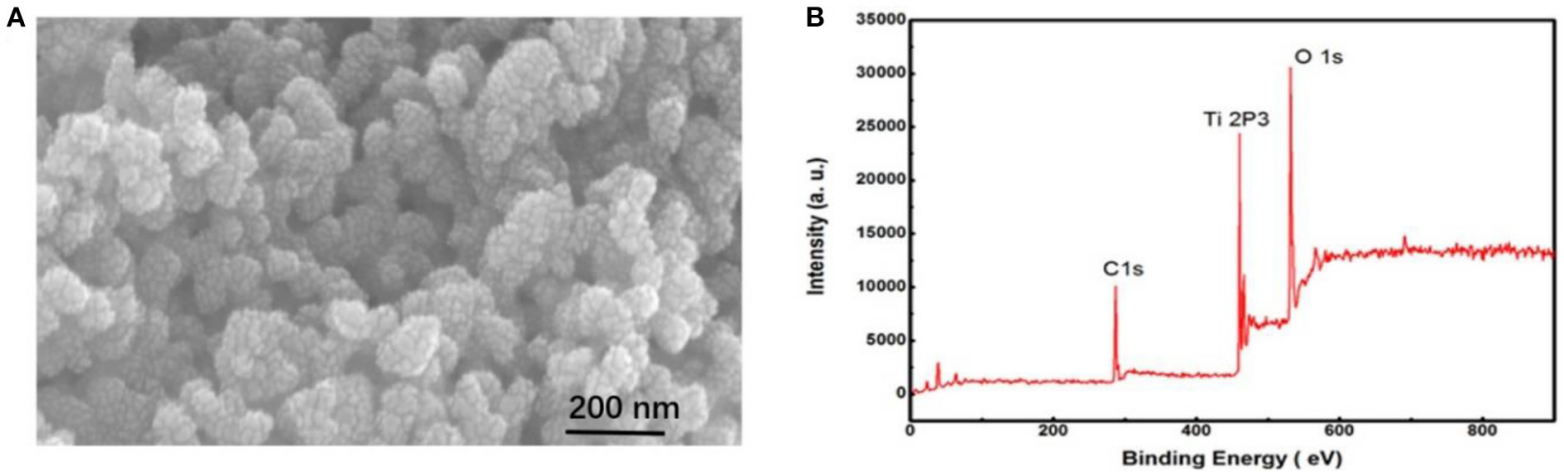
SEM image and XPS characterization of TiO_2_:C-NPs. **(A)** SEM image of TiO_2_:C-NPs; **(B)** XPS characterization of TiO_2_:C-NPs.

Bio-recognition elements (photoelectric active materials) and probes are two necessary devices for current-mode photoelectric sensors. The bio-recognition probes are essentially the same in all types of biosensors (protein or nucleic acid molecules). Therefore, to improve sensor performance, researchers have focused on improving the properties of photoelectric active materials. TiO_2_-NPs are often used as the base material of sensor electrodes because of their high stability, nontoxicity, and high biological safety. Elemental C was doped into TiO_2_-NPs to further improve the photocurrent response. The doped C created new electronic energy states in the mid-gap region of TiO_2_ to promote the flow of photogenerated electrons to the electrode and promote the transfer of photogenerated holes in the CdTe to V_0_ electronic states in TiO_2_ (Scheme 1B, process 3). This effectively reduces the recombination of electron holes in CdTe QDs (30, 31). Scanning electron microscopy (SEM) of TiO_2_:C-NPs (Figure 2A) showed that the average inner diameter of TiO_2_:C-NPs was ~50 nm. Figure 2B shows the XPS spectrum of TiO_2_:C-NPs, again confirming that C was successfully doped into the TiO_2_ NPs.

**Figure 3:**
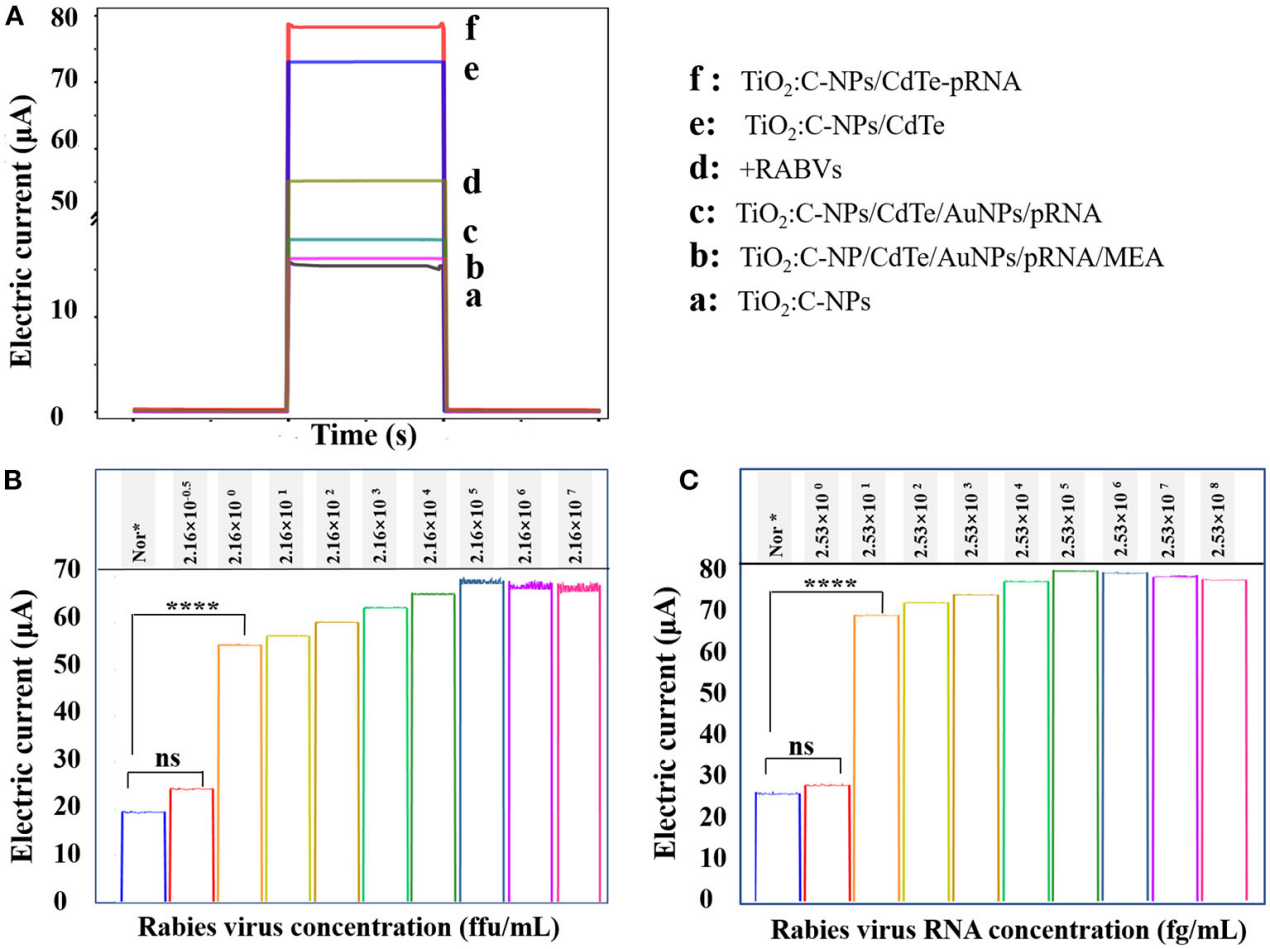
Corresponding photocurrent response of the electrode. **(A)** Corresponding photocurrent response of the electrode: (a) TiO_2_:C-NPs, (b) TiO_2_:C-NP/CdTe/AuNPs/pRNA/MEA, (c) TiO_2_:C-NPs/CdTe/AuNPs/pRNA, (d) +RABVs, (e) TiO_2_:C-NPs/CdTe, and (f) TiO_2_:C-NPs/CdTe-pRNA. Photocurrent response of different concentrations of **(B)** RABV virual particle and **(C)** RNA in cell lysates, each sample was measured five times.

The photocurrent response of each component in the aptasensor was measured (Figure 3A). TiO_2_:C-NPs were used as the base electrode of the sensor and exhibited a relatively low photocurrent response (curve a) due to the limited absorption of ultraviolet light. The photocurrent intensity (curve f) increased by almost five-fold relative to that of the TiO_2_:C-NPs electrode after CdTe QDs immobilization. There are two reasons for this: First, the TiO_2_:C-NPs electrode loaded large quantities of CdTe QDs, and the modified CdTe QDs increased the absorption of the excited light. Second, the doped C inhibited the electron-hole recombination of CdTe QDs. The photocurrent intensity subsequently decreased after NH_2_-RNA was successively immobilized on the CdTe QD-modified electrode (curve e) due to the comparatively weak charge transfer of RNA sequences along with small organic molecules.

The photocurrent dramatically decreased because of exciton energy transfer from the CdTe QDs to the AuNPs after RNA–Au/SH immobilization (curve c). The photocurrent intensity decreased slightly when MEA was immobilized vs. RNA–Au/SH (curve b). The photocurrent intensity significantly recovered when the as-prepared sensing electrode was incubated with 20 μL of 2.16 × 10^1^ ffu/mL RABVs (curve d).

This was because the RABV aptamer was specifically bound with RABVs; meanwhile, RNA–Au/SH was released from the electrode surface, thus leading to a destroyed exciton energy transfer. Therefore, the photocurrent responses resulted in the successful construction of the proposed aptasensor.

The energy receptor RNA Au/SH is released from the electrode surface after the RABV specifically binds to the biometric probe of the sensor. The receptor can then no longer accept the energy generated by excitation. Therefore, energy is transferred to the electrode of the sensor, leading to an increase in the photocurrent of the electrode. This experiment demonstrated that the designed aptamer sensor could be successfully used to detect RABV by measuring the photocurrent of each component of the sensor.

**Figure 4:**
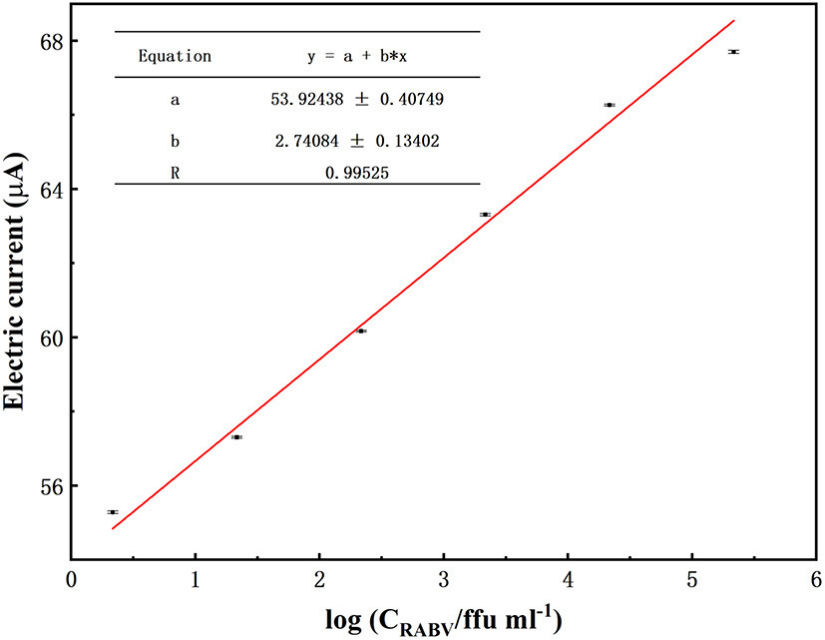
Biosensor response to different concentrations of RABV. Biosensor response toward different concentrations of RABV from 2.16 × 10^5^ ffu/ml to 2.16 ffu/ml. Nor* is a normal NA cell. The error bars indicate the standard deviation of 10 replicates.

The experiments above confirmed efficient exciton energy transfer between the CdTe QDs and AuNPs; thus, they could be used for photoelectrochemical detection of RABV. To verify the detection sensitivity of the sensor, RABV was next used as the target analyte for detection *via* the photoelectrochemical sensing platform. The results showed that the photocurrent intensity was 55.16 μA, 57.191 μA, 59.49 μA, 63.17 μA, 66.10 μA, 67.59 μA, 66.65 μA, and 66.51 μA when the virus concentration was 2.16 ffu/ml to 2.16 × 10^7^ ffu/ml (Figure 3B). From 2.16 × 10^0^ ffu/mL to 2.16 × 10^5^ ffu/mL, the photocurrent was linear with the logarithm of the target concentration. The regression equation was I = 54.6749 + 2.4651 logC (*R* = 0.99525) (Figure 4). The minimum detection concentration limit for RABV is ~2.16 ffu/ml—this is 1–3 orders of magnitude higher than the recently reported detection sensitivity for RABV (32). To ensure the reliability of the detection limits of PBA, the experiment on the detection limits of PBA using RABV RNA concentration (fg/mL) (Figure 3C) have been done. The results indicated similar detection limits by both RABV concentration (ffu/mL) (Figure 3B) and RABV RNA concentration (fg/mL) (Figure 3C). The 2.53 × 10^1^ fg/mL of RABV RNA concentration (fg/mL) showed 68 μA electric current, which is 45 μA higher than that of the control and demonstrated obvious difference (Figure 3C). The sensor has a high sensitivity for the specific detection of rabies, and the detection limit concentration for RABV detection has greater advantages than other detection platforms.

**Figure 5:**
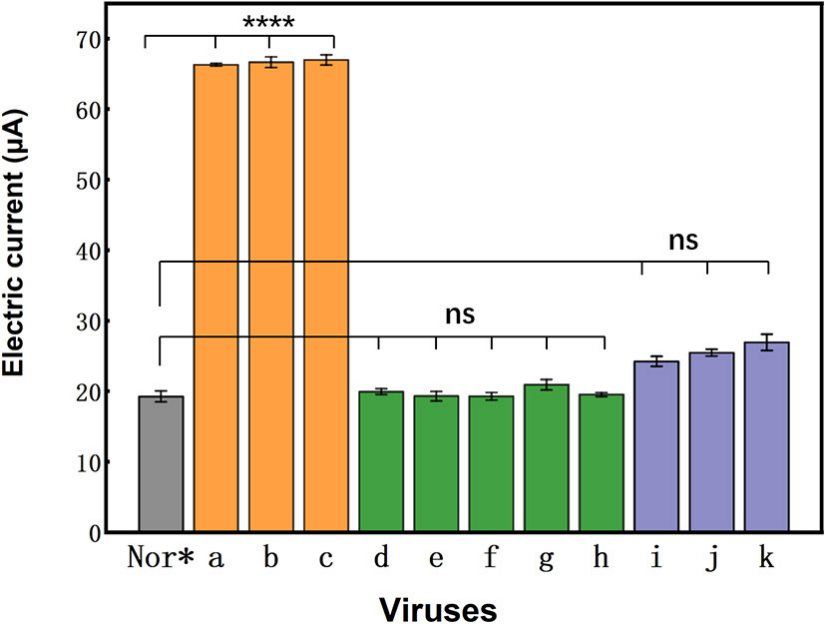
Specificity of the photoelectrochemical biosensor approach to different viruses. Photocurrent responses of the universal aptasensor to 2.16 × 10^4^ ffu/ml of normal NA cell (Nor*), rRC-HL (a), CVS-24 (b), GX074 (c), CSFV (d), PRRSV-5 (e), PRRSV-96 (f), PCV (g), and VSV-g (h), and photocurrent responses of the p-r-RNA aptasensor, p-w-RNA aptasensor, and p-c-RNA aptasensor to 2.16 × 10^4^ ffu/ml of rRC-HL (i), CVS-24 (j), and GX074 (k).

Specificity is an important indicator for nucleic acid sensors because non-specific adsorption affects the sensitivity and accuracy of sensor detection. To demonstrate that the photocurrent response was due to the specific binding of the probe to the pathogen nucleic acids, representative pathogen nucleic acids, such as CSFV, PRRSV-5, PRRSV-96, PCV, and VSV-g, were selected as interferants. The results showed that CSFV, PRRSV-5, PRRSV-96, PCV, and VSV-g had basically no effect on the nucleic acid sensors' photocurrent response (Figure 5). We have carried out five repeated experiments (Supplementary Table 1 and Supplementary Figure 1), and the results showed that there are little differences between experimental groups. It shows that the PBA system is a very stable and advantageous detection method. Thus, this probe is a universal probe for rabies and does not respond to other viruses. The nucleic acid sensor designed here had good specificity with no current changes with non-specific adsorption.

**Figure 6:**
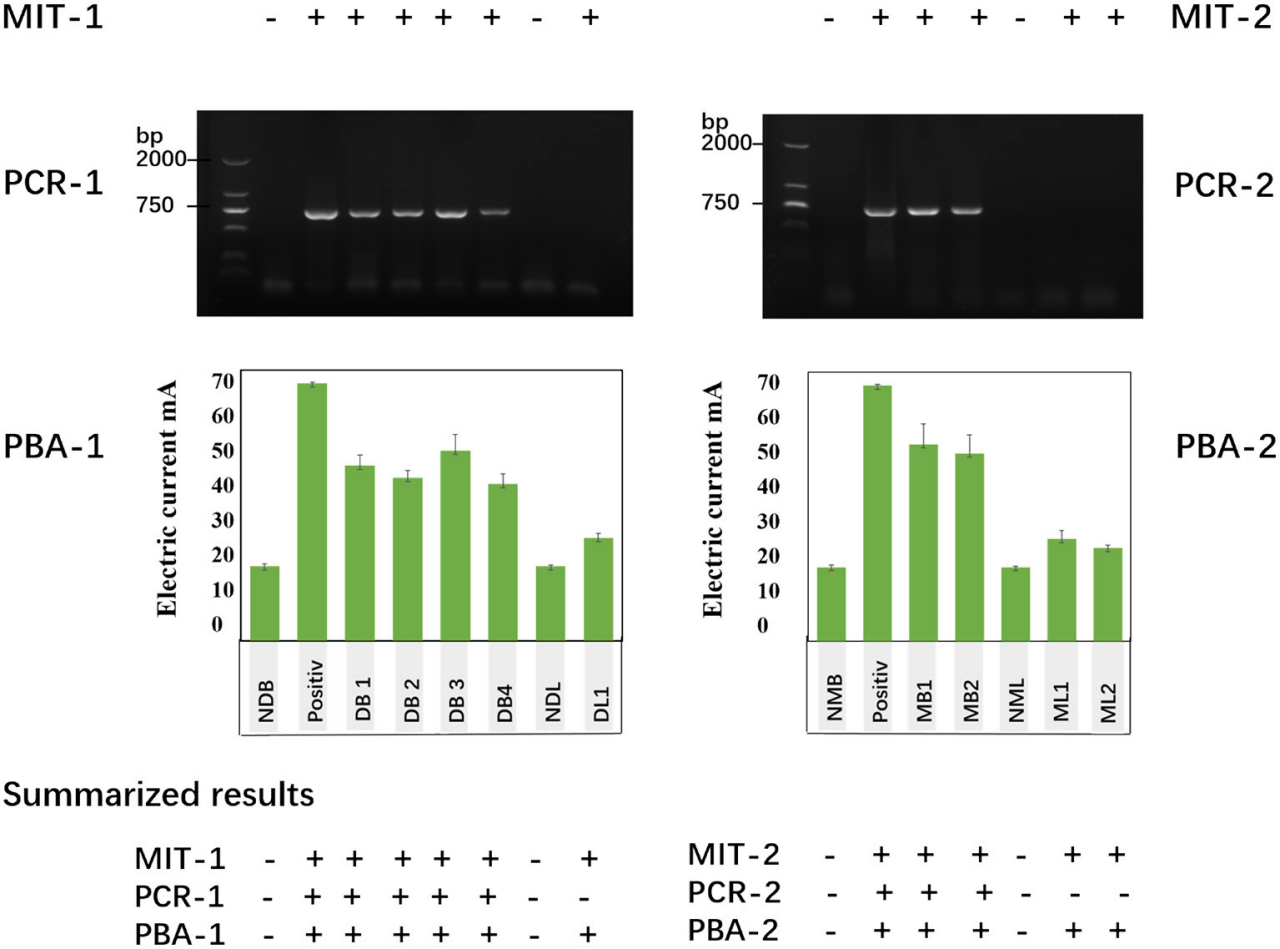
Tissue samples were detected using MIT, RT-PCR, and a photoelectrochemical biosensor approach (PBA). MIT-1, PCR1, and PBA1 represents the detection results from dog brain and lung samples. Positive indicates the positive control from the standard rabies virus strain (CVS-24)-infected cell samples. The other samples include brain samples of four dogs (DB1, DB2, DB3, and DB4), a normal dog lung sample (NDL), and a dog lung sample (DL1, the same dog of DB1). MIT-2, PCR2, and PBA2 showed the detection results from mouse brain and lung samples. Positive indicates the standard rabies virus strain (CVS-24)-infected cell samples, two mouse brain samples (DM1, DM2), a normal mouse lung (NML), and two mouse lung samples (ML1, ML2).

We evaluated the brain and lung samples from rabid dogs as well as rodent models of rabies using PBA. We simultaneously measured the same samples by MIT and RT-PCR. The results indicated that the degree of sensitivity by PBA was far better than that by RT-PCR. The positive brain samples detected by RT-PCR could also be detected as positive by PBA: DB1 (45.37 ± 2.61 μA), DB2 (42.17 ± 1.83 μA), DB3 (49.17 ± 4.15 μA), and DB4 (40.54 ± 2.62 μA). However, three lung tissue samples were negative by RT-PCR but positive by an increase in the electric current: and they are a dog lung sample (DL1: 26.55 ± 1.23 μA) and two mouse lung samples (ML1: 26.53 ± 2.12 μA, ML2: 26.53 ± 2.12 μA). So, we found that PBA has delivered 100% diagnostic specificity and sensibility for rabies diagnosis as MIT (Figure 6).

## 5. Discussion

In this study we successfully constructed a new detection method for RABV diagnosis based on PBA. According to the detection results of RABV, we found that the PBA method showed a simple device, low cost and easy miniaturization. It indicated high specificity, differentiated RABV and VSV-g, CSFV, PRRSV-5, PRRSV-96, and PCV (Figure 5), and high sensitivity and can detect as low as 2.16 × 10^0^ ffu/mL RABV. By comparing the results of PBA with that of a conventional/traditional MIT and a modern genetic RT-PCR method, the PBA indicated a certain degree of comparative advantage (Figure 6). Our PBA method also showed 1–3 orders of magnitude higher than a fluorescence immunosorbent assay (FIA) for the detection of RABV G protein based on quantum dot labeling. FIA detection limit was 6.25 × 10^3^ ffu/mL (32)while our PBA is 2.16 ffu/mL. Regarding the specificity of PBA against RABV-related viruses, we could not accurately test those related viruses in this study because the related viruses are not available. PBA can be combined with nested PCR to resolve the specificity problem and avoid yielding false positive result.

Here, CdTe QDs and AuNCs were the energy transfer donors and acceptors, respectively. As an energy donor, CdTe QDs have good emission spectrum regulation, and their emission spectrum range can be calibrated *via* the Cd:Te ratio. The CdTe QDs were 2.93 nm when the molar ratio of Cd:Te was 1:0.1. The UV-visible absorption peak was 510 nm, and the emission peak was 560 nm. The absorption peak of the AuNPs energy receptor is 538 nm.

This rabies sensor started with CdTe AQDS as powerful energy donors adsorbed onto the electrode surface of carbon-doped titanium dioxide nanomaterials (TiO_2_:C-NPs) *via* layer-by-layer assembly. The NH_2_-RNA-SH-AuNPs were then immobilized on the surface of the TiO_2_:C-NPs/CdTe electrode by the EDC/NHS coupling reaction between the amino and carboxyl groups. The sensor electrode was successfully constructed by blocking the active sites of the electrode with MEA. Here, the exciton of CdTe QDs and SPR effect of AuNCs was simultaneous, thus resulting in fast and efficient energy transfer.

In this paper, three experimental methods were used to detect the same samples. First, MIT is sensitive and has a high accuracy rate. Secondly, the Fluorescence method was used for additional identification of specificity. Third, PBA detection showed many more advantages, such as shorter detection time, lower price, higher accuracy and lower detection limits. The core components of PBA detection include the fabrication of the sensor, the components of the sensor are shown in Scheme 1A, including two layers of material on the ITO. Each sensor can test one sample, and it only costs ¥ 0.2 yuan from construction to obtaining the test results. It's important that the result could be shown in < 5 min, and the efficiency is very high; as compared to RT-PCR detection. If one kit of RT-PCR can measure 100 samples, the price of one sample is almost ¥ 20~30 yuan, while the results from total RNA extraction to cDNA synthesis and then PCR and electrophoresis respectively one after the other took almost 5 h to show results. Most importantly, its detection limit is so attractive that the minimum detection concentration limit for rabies virus is ~2.16 ffu/mL. We consulted the relevant bibliography, which reported the detection methods for RABV (dFAT, MIT, RT-PCR, Nested RT-PCR, RT-qPCR and LFD) and compared their detection limits with PBA (Supplementary Table 2). Although the PBA showed some advantages to a certain extent, the other detection methods are still useful till now.

Here, the resulting electrode aptamer is a universal probe of RABV, and the PBA detection can detect it, regardless of the virus strain, as long as it is a RABV. However, as a universal probe, it cannot distinguish which strain of RABV it is. We used this platform to establish a detection mechanism, which gave us a good inspiration and a reasonable probe design that can be used to distinguish different RABV strains in the future. The PBA platform for sample detection presents the best option, and the detection range is 2.16 ffu/mL to 2.16 × 10^5^ ffu/mL, showing a reliable linear regression (*R* = 0.99525). Therefore, the detection results are reliable and innovative. The platform can detect brain and lung tissue samples or cell culture samples with RABV infection. The photocurrent from the brain samples increased more than two-fold vs. samples without RABV infection. Some lung tissue samples detected as negative by RT-PCR were positive by PBA with an increased photocurrent. And once the aptasensor is constructed, the subsequent results can be displayed within 5 min, the whole process is convenient and fast. The successful construction of this diagnostic platform broadens the available test methods for various sample bioanalysis and provides a theoretical and practical basis for the establishment of other bioanalytical detection methods.

## 6. Conclusion

In conclusion, CdTe QDs can transfer energy donors that have a tunable extinction coefficient and high luminous intensity. This can significantly increase the number of photogenerated electron pairs. AuNPs have a strong absorption band and can absorb the energy excited by electron-hole pairs, thus reducing the number of electrons in the electrode. More importantly, the overlapping emission and absorption spectra between the CdTe QDs and AuNPs and the distance between them satisfied the necessary conditions for efficient energy transfer.

An aptamer-sensing platform for rabies was constructed based on the above principles. The sensor offered a low detection limit, wide linear detection range, good selectivity, reproducibility, and good stability. The sensor offered specific detection of rabies virus with value in diagnostics. The platform can detect viral RNA from the rabies virus virions in cell culture supernatant, in brain and lung tissue samples, and in cultured cells. This tool may help alleviate the complex and tedious traditional rabies virus diagnosis methods.

## Data availability statement

The original contributions presented in the study are included in the article/Supplementary material, further inquiries can be directed to the corresponding authors.

## Ethics statement

The animal study was reviewed and approved by the Biological Safety Laboratory of Guangxi Center for Animal Diseases Control and Prevention.

## Author contributions

Conceptualization: TL, ZH, X-NL, and Y-JL. Methodology: X-NL, ZH, and Y-JL. Formal analysis and writing—original draft preparation: Y-JL. Investigation: Y-JL, D-DL, Z-LC, YC, D-LY, and H-YZ. Resources and supervision: ZH, X-NL, and TL. Writing—review and editing: Y-JL, YC, and AK. Funding acquisition: TL and ZH. All authors contributed to the article and approved the submitted version.
