# Supplementary figures and images for: Detection of rabies virus via exciton energy transfer between CdTe quantum dots and Au nanoparticles
Source: Front Vet Sci. 2022 Dec 22;9:1079916. doi: 10.3389/fvets.2022.1079916 (PMC9813487; doi:10.3389/fvets.2022.1079916)

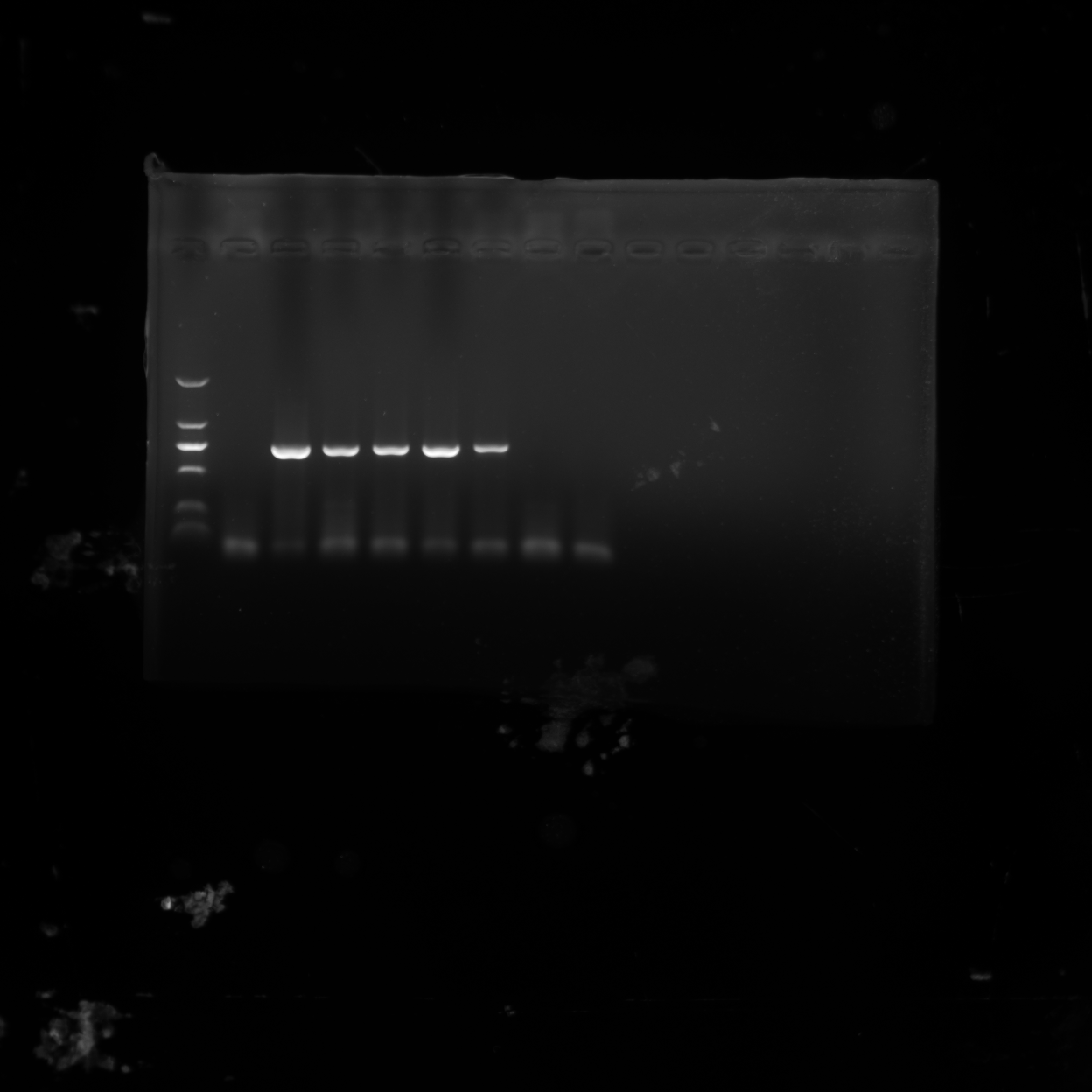

Supplement: Supplementary file 1 [file Data_Sheet_1.ZIP › Raw data/Figure 6/Figure 6 PCR-1.tif]

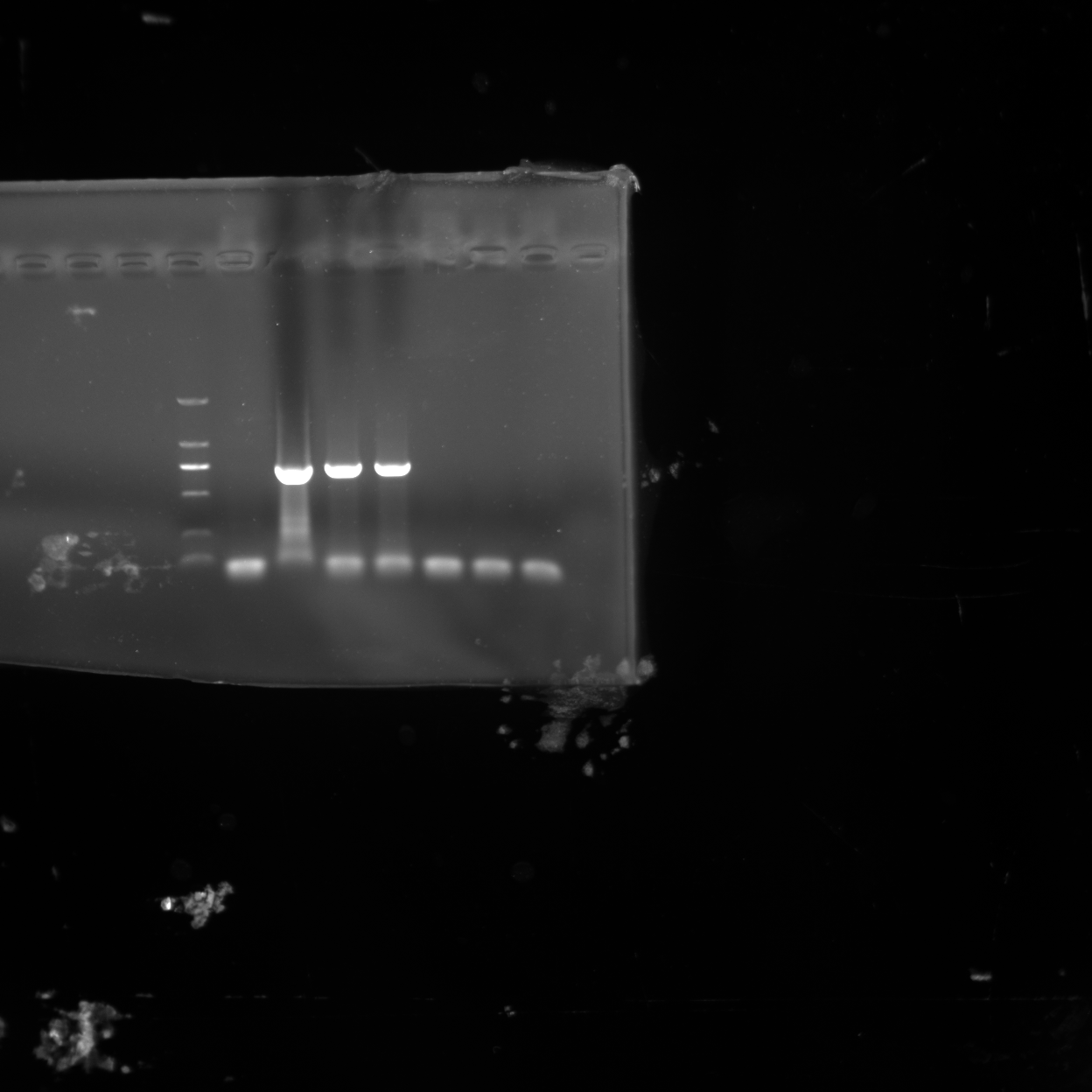

Supplement: Supplementary file 1 [file Data_Sheet_1.ZIP › Raw data/Figure 6/Figure 6 PCR-2.tif]

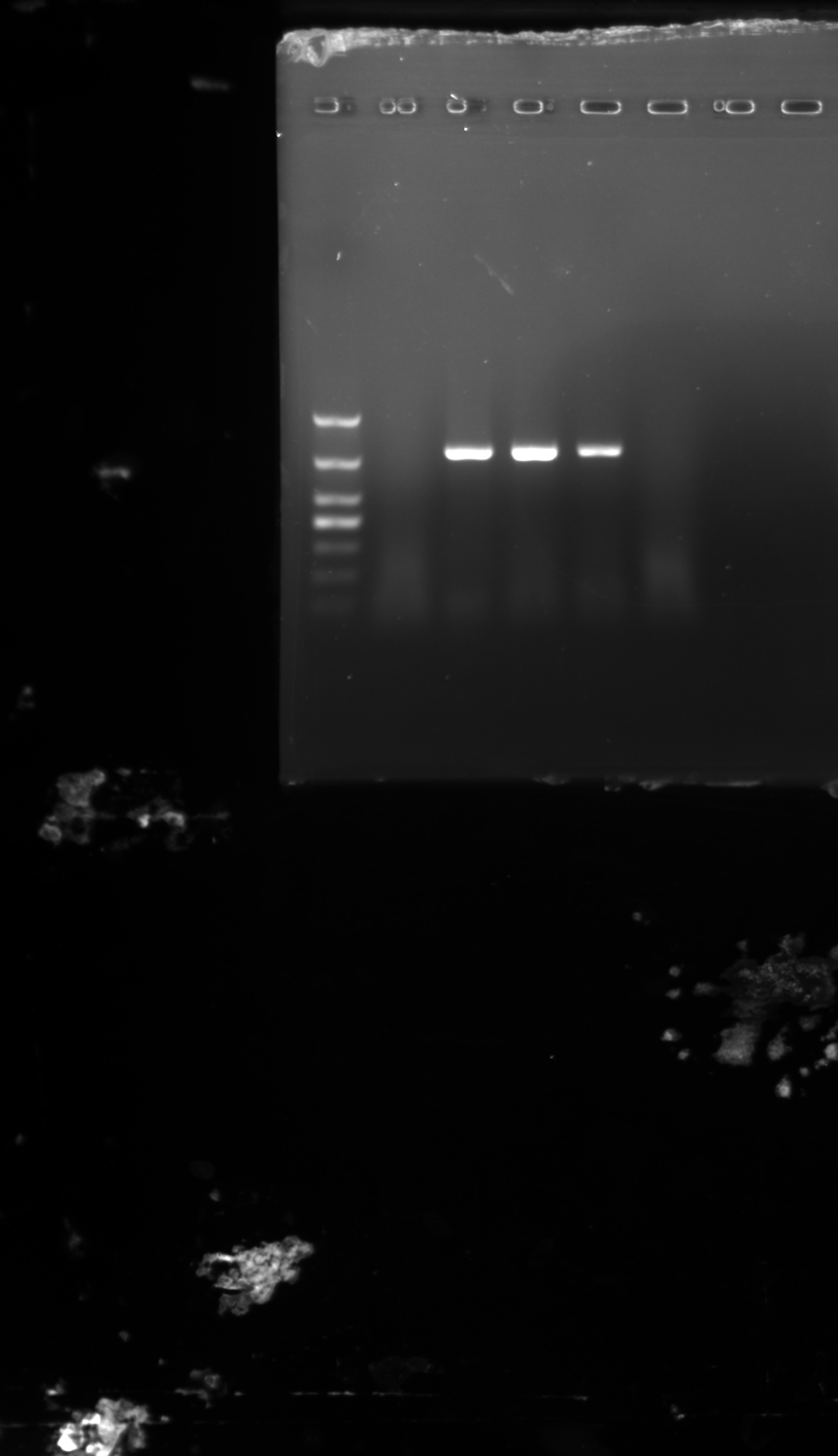

Supplement: Supplementary file 2 [file Data_Sheet_2.ZIP › Explanation of the raw image for Figure 6 PCR-2/Figure 2.tif]

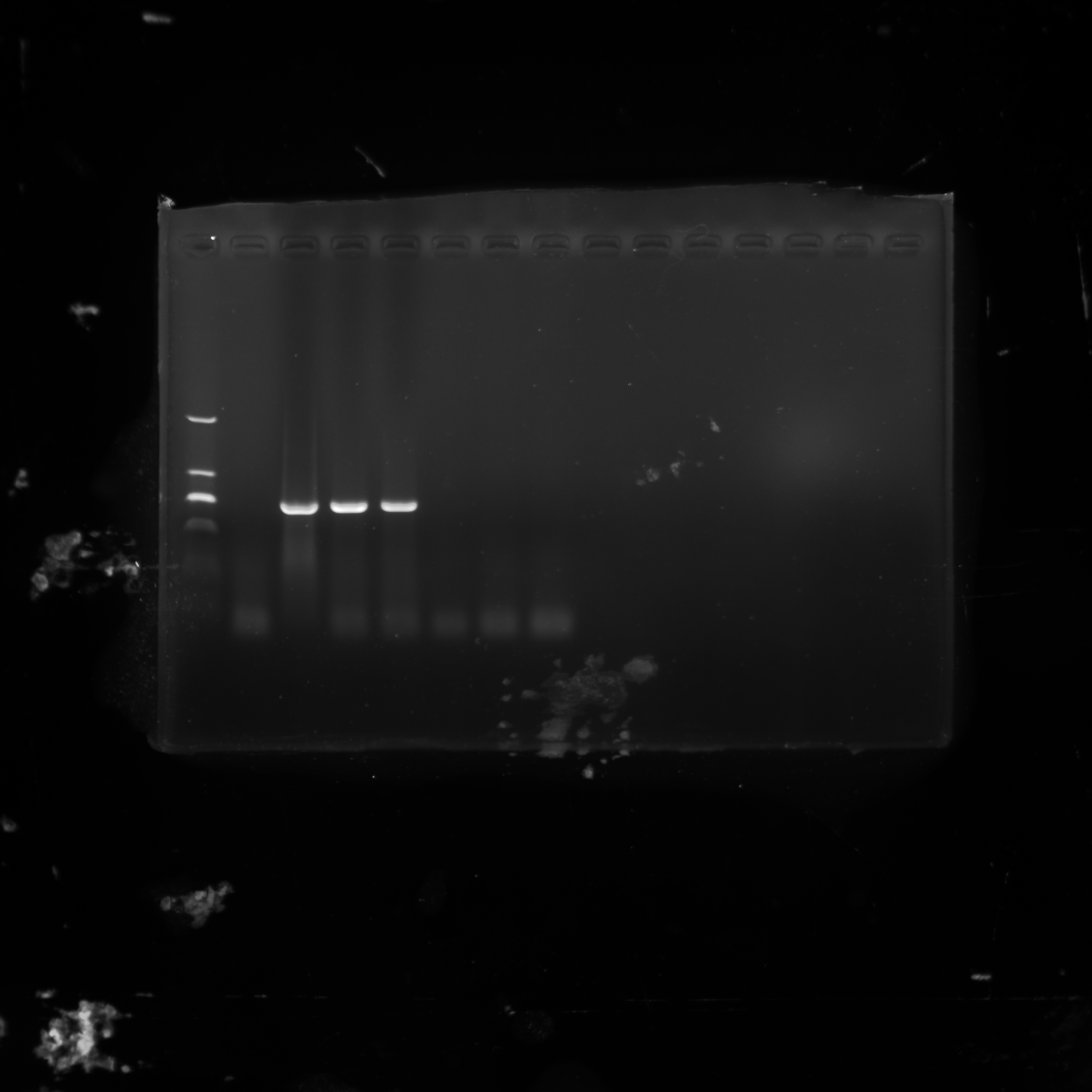

Supplement: Supplementary file 2 [file Data_Sheet_2.ZIP › Explanation of the raw image for Figure 6 PCR-2/Figure 3.tif]
