# Supplementary material for: Detection of rabies virus via exciton energy transfer between CdTe quantum dots and Au nanoparticles
Source: Front Vet Sci. 2022 Dec 22;9:1079916. doi: 10.3389/fvets.2022.1079916 (PMC9813487; doi:10.3389/fvets.2022.1079916)
Supplement: Supplementary file 3 [file Table_1.DOCX]

**Supplementary Table 1 Specificity of the photoelectrochemical biosensor approach to different viruses with five experiments**

| **Viruses**  **Statistics** | **Nor*** | **a** | **b** | **c** | **d** | **e** | **f** | **g** | **h** | **i** | **j** | **k** |
| --- | --- | --- | --- | --- | --- | --- | --- | --- | --- | --- | --- | --- |
| **Mean(μA)** | 19.26 | 66.28 | 66.66 | 66.99 | 19.81 | 19.31 | 19.30 | 20.93 | 19.56 | 24.24 | 25.48 | 26.94 |
| **Standard Deviation** | **0.75** | **0.19** | **0.79** | **0.75** | **0.34** | **0.66** | **0.56** | **0.77** | **0.32** | **0.71** | **0.50** | **1.15** |


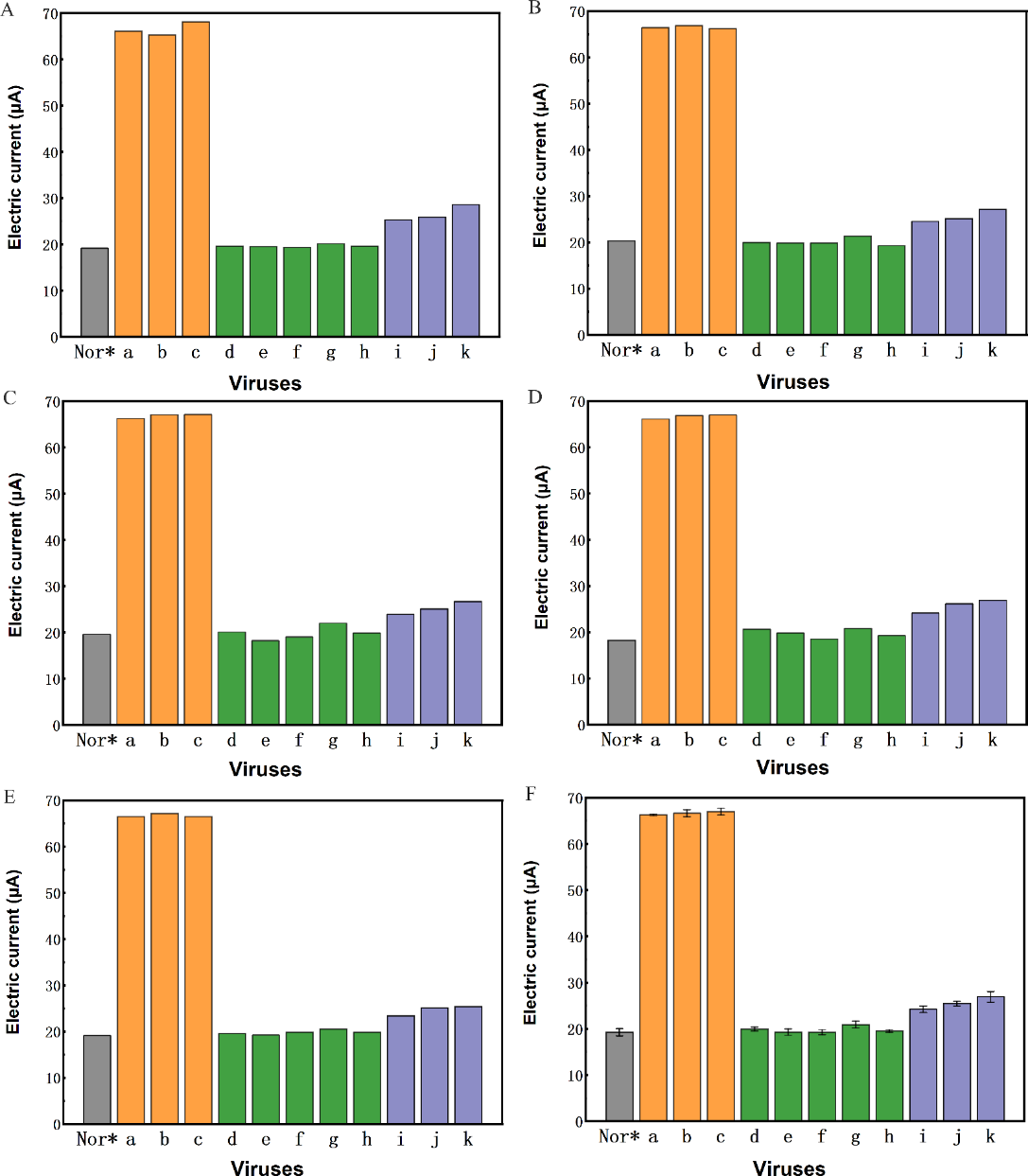


**Supplementary Figure 1** Specificity of the photoelectrochemical biosensor approach to different viruses with five experiments (Figures A to E were the 1st to 5th experiments respectively, Figures F Figure F is a statistical summary of the results of 5 experiments). Photocurrent responses of the universal aptasensor to 2.16🞨10^4^ ffu/ml of normal NA cell (Nor*), rRC-HL (a), CVS-24 (b), GX074 (c), CSFV (d), PRRSV-5 (e), PRRSV-96 (f), PCV (g), and VSV-g (h), and photocurrent responses of the p-r-RNA aptasensor, p-w-RNA aptasensor, and p-c-RNA aptasensor to 2.16🞨10^4^ ffu/ml of rRC-HL (i), CVS-24 (j), and GX074 (k).

**Supplementary Table 2 A comparison of the proposed system for rabies virus detection**

| Ref. No. | Detection method | Analyte | sensitivity | specificity | accuracy | Linear range | Detective limit | Reference |
| --- | --- | --- | --- | --- | --- | --- | --- | --- |
|  | PBA | rabies virus RNA | 100% | 100% | 100% | 2.16×10^0^ to 2.16×10^5^ ffu/mL | 2.16 ffu/mL or  25.3 fg/mL | **This work** |
| 1 | dFAT | rabies virus antigen | 93.58% | 95.9% | 95.67% | / | / | [1] |
| 2 | MIT | Ratio of mouse death | 89.58% | 95.9% | 98.97% | / | / | [2] |
| 3 | RT-PCR | rabies virus RNA | 100% |  |  | / | 0.00002 TCID_50_/ml | [3] |
| 4 | Nested RT-PCR | rabies virus RNA | 100% | 100% | 100% | 2.24×10^0^ to 2.24×10^5^ molecules/μL | 2.24×10^0^ molecules/μL | [4] |
| 5 | RT-qPCR | rabies virus RNA | 100% | 97.6% | 100% | / | 10 copies/μL | [5] |
| 6 | hnRT-PCR | rabies virus RNA | 100% | 100% | 100% | 10^1^ to 10^10^ copies/μL | 10 copies/μL | [6] |
| 7 | LFD | rabies virus RNA | 88%-98% | 100% | 100% | / | / | [7] |

**References**

[1] N.H.F. Centoamore, M.E.R. Chierato, V.B.V. Silveira, K.M. Asano, K. Iamamoto, W.O. Fahl, K.C. Scheffer, S.M. Achkar, L.P. Mesquita, P.C. Maiorka, E. Mori, Comparison of five different laboratory techniques for the rabies diagnosis in clinically suspected cattle in brazil, J. Virol. Methods 283 (2020) 113918, https://doi.org/10.1016/j.jviromet.2020.113918.

[2] A.C. Rodrigues, R.M.N. Marcusso, D.N. Souza, W.O. Fahl, G.M.M. Caporale, C.I. Macedo, J.G. Castilho, A comparative study of direct fluorescent antibody, mouse inoculation, and tissue culture infection testing for rabies diagnoses, J. Virol. Methods 300 (2022) 114426, https://doi.org/10.1016/j.jviromet.2021.114426.

[3] J.E. Whitby, P.R. Heaton, H.E. Whitby, E. O'Sullivan, P. Johnstone, Rapid detection of rabies and rabies-related viruses by rt-pcr and enzyme-linked immunosorbent assay, J. Virol. Methods 69 (1-2) (1997) 63-72, https://doi.org/10.1016/s0166-0934(97)00143-2.

[4] Y. Wang, W. Xu, H. Guo, W. Gong, B. He, Z. Tu, C. Tu, Y. Feng, Evaluation of a universal nested reverse transcription polymerase chain reaction for the detection of lyssaviruses, J. Vis. Exp. (147) (2019), https://doi.org/10.3791/59428.

[5] V. Suin, F. Nazé, A. Francart, S. Lamoral, S. De Craeye, M. Kalai, S. Van Gucht, A two-step lyssavirus real-time polymerase chain reaction using degenerate primers with superior sensitivity to the fluorescent antigen test, Biomed Res. Int. 2014 (2014) 1-12, https://doi.org/10.1155/2014/256175.

[6] J. Coertse, J. Weyer, L.H. Nel, W. Markotter, Improved pcr methods for detection of african rabies and rabies-related lyssaviruses, J. Clin. Microbiol. 48 (11) (2010) 3949-3955, https://doi.org/10.1128/JCM.01256-10.

[7] K. Kimitsuki, N. Saito, K. Yamada, C. Park, S. Inoue, M. Suzuki, M. Saito-Obata, Y. Kamiya, D.L. Manalo, C.S. Demetria, M.R. Mananggit, B.P. Quiambao, A. Nishizono, Evaluation of the diagnostic accuracy of lateral flow devices as a tool to diagnose rabies in post-mortem animals, Plos Neglect. Trop. Dis. 14 (11) (2020) e8844, https://doi.org/10.1371/journal.pntd.0008844.
