# Supplementary material for: Detection of rabies virus via exciton energy transfer between CdTe quantum dots and Au nanoparticles
Source: Front Vet Sci. 2022 Dec 22;9:1079916. doi: 10.3389/fvets.2022.1079916 (PMC9813487; doi:10.3389/fvets.2022.1079916)
Supplement: Supplementary file 2 [file Explanation of PCR-2.pdf › Explanation of the raw image for Figure 6 PCR-2/Explanation of PCR-2.pdf]

Explanation of the raw image for Figure 6 PCR-2

Dear editor,

Thank you for your careful review.

We have carried out 3 repeated experiments for PCR-2,and the original images for gels as follow (Fingure 1 to Fingure 3), and the results showed that there are almost no difference between each experiment. It shows that our experimental results are reliable .

But unfortunately, the experimental gel map uploaded last time was just one of them (Fingure 6-1), and we indeed put the other (Figure 6- 3) in the main article. Now we make the corrections,and upload all original images for you . Thank you very much!

Sincerely yours

Ting Rong Luo

Phone: +086-771-3232248;

Email: gx_3267030@163.com/ tingrongluo@gxu.edu.cn

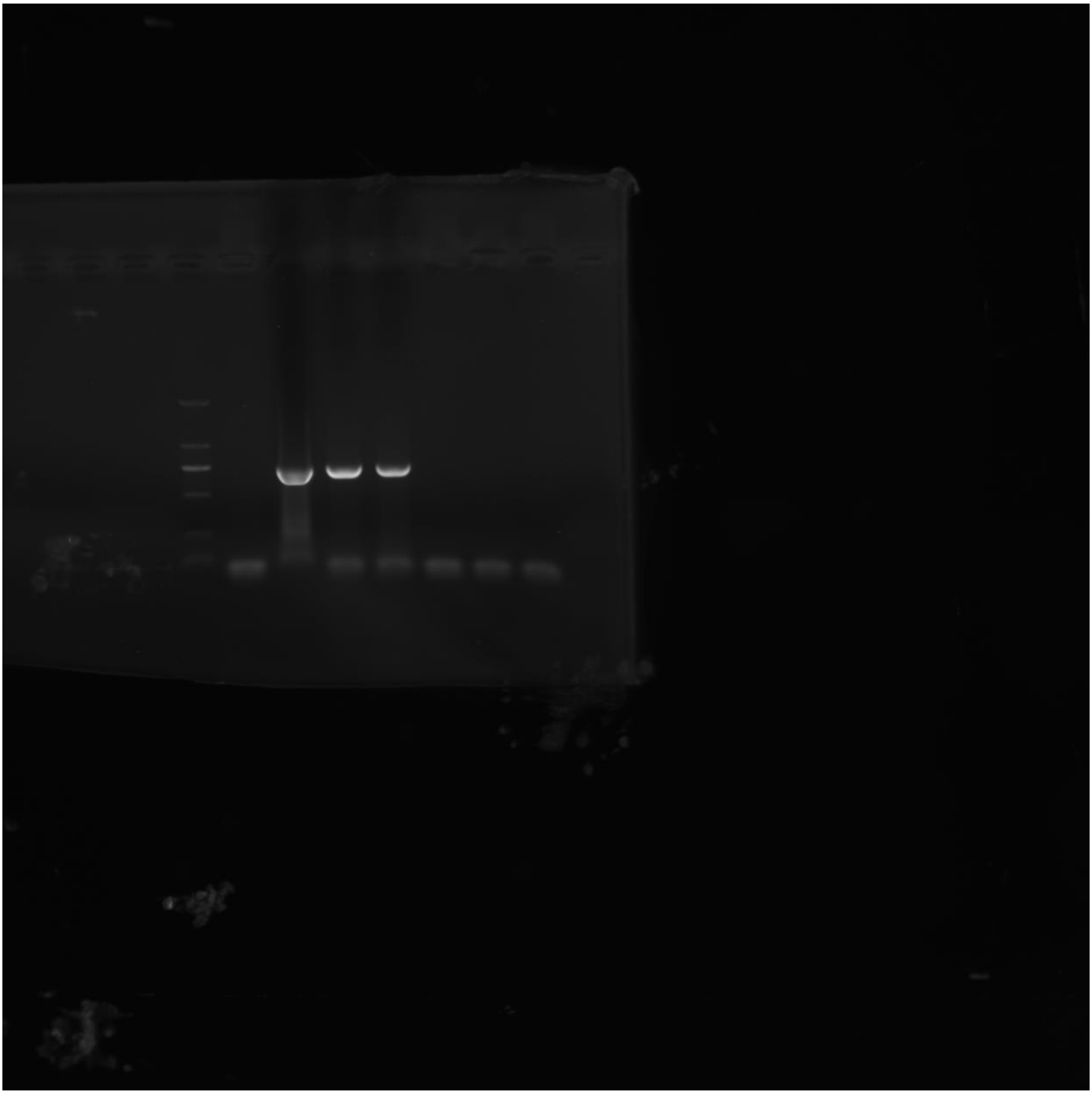

Figure 6-1

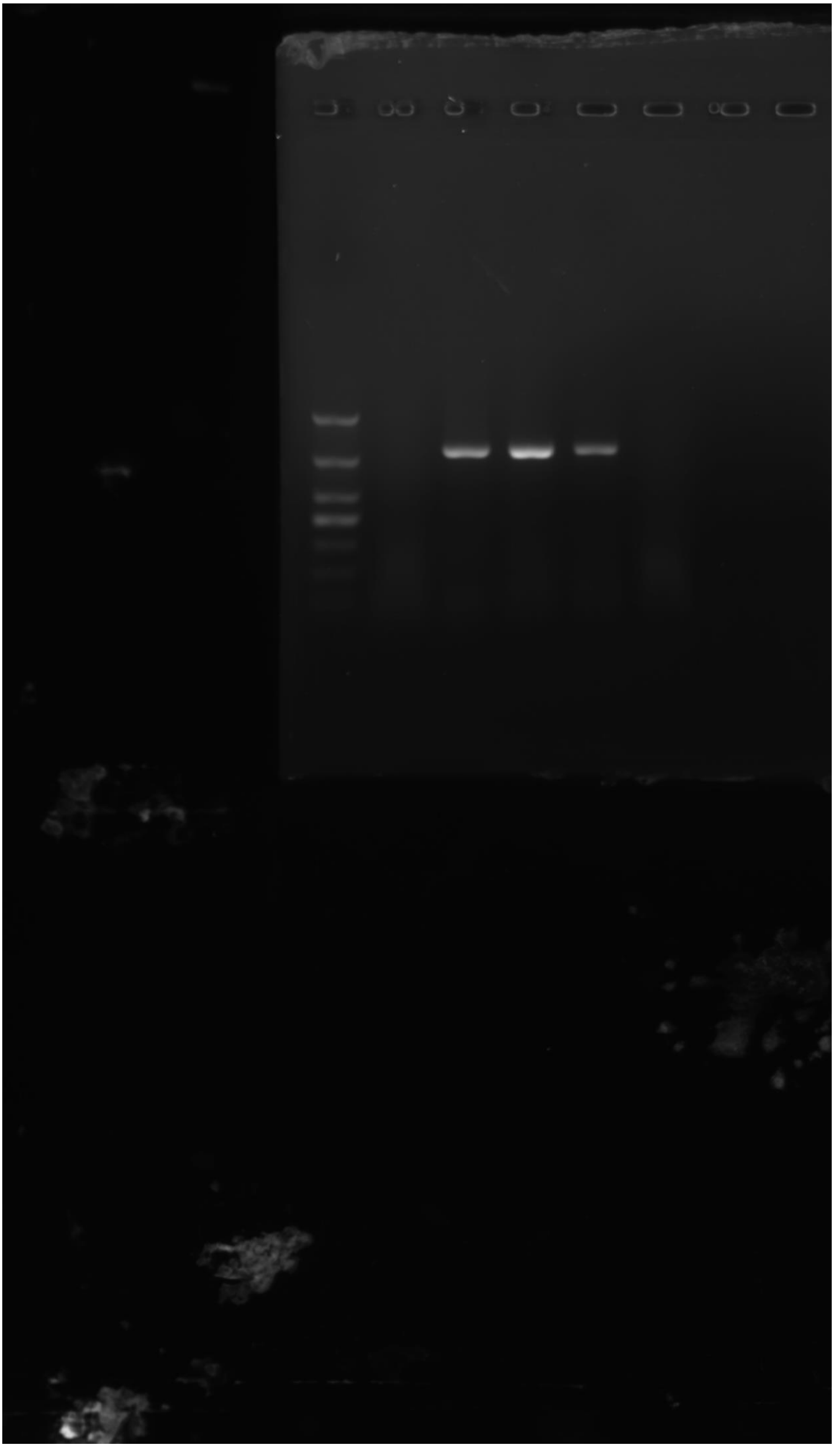

Figure 6-2

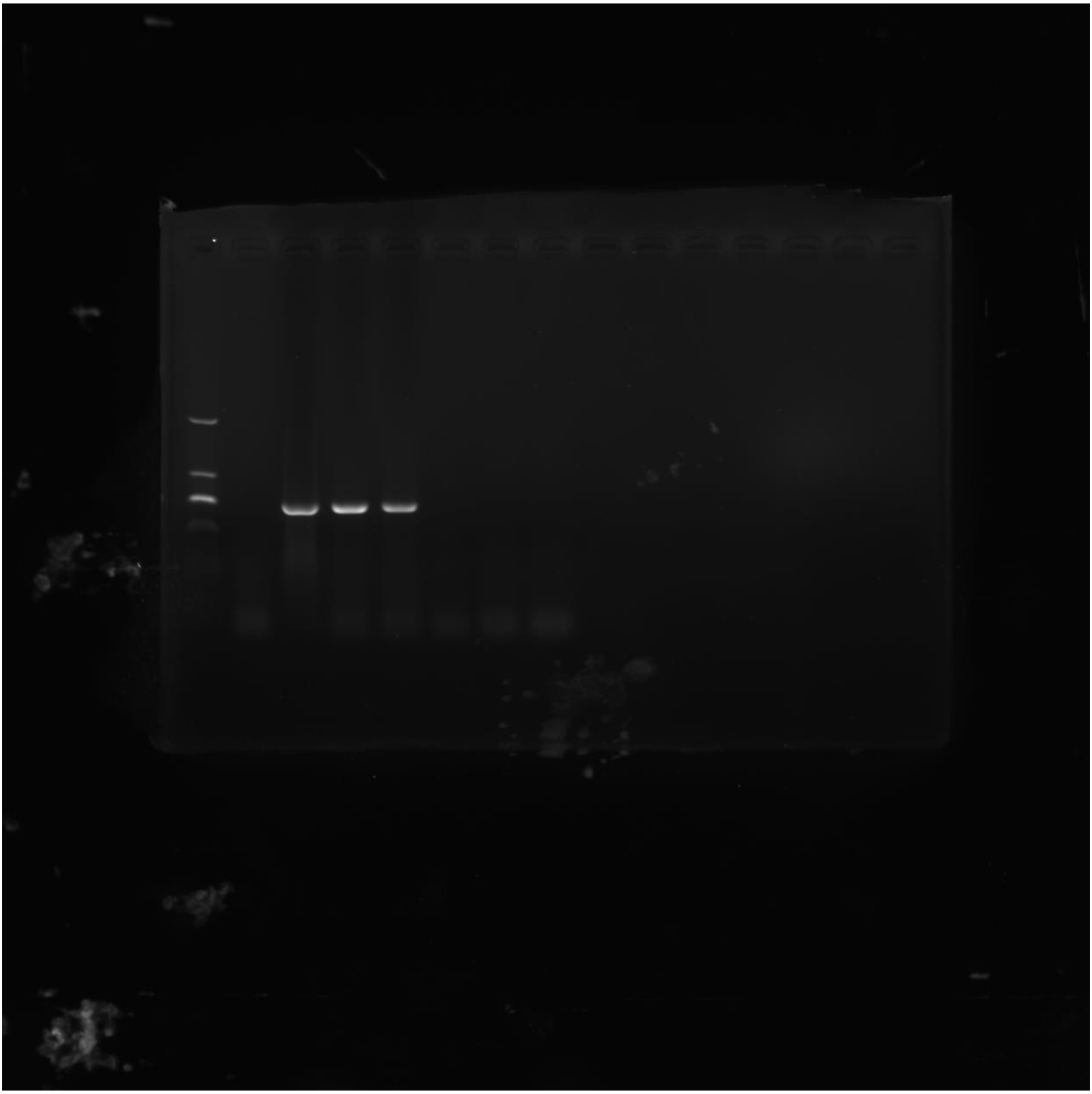

Figure 6-3
